# Cecal microbiota and *Clostridium perfringens* in broilers fed barley-based diets: Effects of enzyme supplementation and degree of grinding

**DOI:** 10.1016/j.psj.2026.106543

**Published:** 2026-01-28

**Authors:** Eva Lena Estensmo, Camilla Sekse, Franciska S. Steinhoff, Kari Ljøkjel, Khaled Itani, Muhammad Ahmad, Cathrine Arnason Bøe, Siri Kulberg Sjurseth, Silje Granstad

**Affiliations:** a Department of Animal Health, Welfare and Food Safety, The Norwegian Veterinary Institute, Ås, Norway; b Felleskjøpet Fôrutvikling, Trondheim, Norway; c Department of Animal and Aquacultural Sciences, Norwegian University of Life Sciences, Ås, Norway; d Nortura SA, Oslo, Norway

**Keywords:** Broiler, Microbiota, *Clostridium perfringens*, Barley, NSPase

## Abstract

Barley has been proposed as a promising and more sustainable alternative to common cereals in poultry diets. However, the use of barley in poultry diets has historically been limited, mainly due to observed negative impact on gastrointestinal health and performance. In this study, we explored the potential of incorporating barley into broiler diets, focusing on effects on *Clostridium perfringens*, the causative agent of necrotic enteritis in poultry, and the cecal microbiota. The study included four diets containing 54% barley, with or without the addition of non-starch polysaccharide-degrading enzyme (NSPase), and one control diet without barley. The main ingredients were ground to either a fine or coarse particle size. Low *C. perfringens* levels were maintained in the intestines of broilers fed barley-based diets, suggesting that barley did not predispose to necrotic enteritis in this study. Broilers fed the coarse barley-based diet with NSPase exhibited the lowest abundance of *Lactobacillus* and the highest abundance of *Faecalibacterium*, and their cecal microbiota resembled that of the group fed the control diet. In contrast, broilers fed the coarse barley-based diet without NSPase exhibited the highest abundance of *Lactobacillus* among all groups in this study, along with a lower abundance of *Faecalibacterium*. Among the groups that received diets with finely ground barley, regardless of NSPase supplementation, there was no clear shift in the abundance of the two bacterial genera. These observations suggest that feed particle size and NSPase supplementation influence the composition of the cecal microbiota in broilers fed barley-based diets, and that these factors could be utilized as tools to mitigate undesirable health effects associated with the inclusion of barley in poultry diets. The findings of this study highlight the potential of including increased levels of barley in broiler diets without compromising health or performance. Further studies are warranted to explore the effects of similar inclusion levels of different barley varieties on gastrointestinal health and microbiota under varied environmental conditions.

## Introduction

Broiler meat production is among the animal-based food production systems with the lowest environmental footprint (Poore and Nemecek, 2018). Thus, broiler meat production is considered an important contributor to sustainable food production. However, due to unfavorable climatic conditions in Northern Europe, a large proportion of broiler diets in this region consists of imported feed ingredients. In contrast to cereals such as corn, barley has significant production potential in this climate zone and would be a more sustainable alternative.

The nutritional value of barley for broilers is well known (Svihus and Gullord, 2002; Jacob and Pescatore, 2012). Nevertheless, high inclusion levels of barley have not been widely used in broiler diets due to the antinutritive effects of soluble fibers. Soluble non-starch polysaccharides (NSPs) dissolve in water to form viscous, gel-like solutions that increase the viscosity of the digesta in the gut (Choct, 2006). Higher viscosity can impede nutrient absorption and limit the interaction between digestive enzymes and feed components. Additionally, the slower transit time resulting from increased viscosity, combined with the availability of undigested nutrients and easily fermentable fibers, can promote the proliferation of harmful bacteria such as *Clostridium perfringens*. This may lead to imbalances in the gut microbiota, sticky droppings, poor litter quality, and the development of necrotic enteritis (Hofshagen and Kaldhusdal, 1992; Annett et al., 2002; Van Immerseel et al., 2004). Adding fiber-degrading enzymes to broiler diets, such as non-starch polysaccharide-degrading enzyme (NSPase), and the development of new barley varieties leading to lower digesta viscosity, have significantly mitigated this problem, though it has not been eliminated. Several recent experiments have indicated that varied levels of barley (26 – 34%) can be included in the diets without negatively impacting performance (Craig et al., 2019; Perera et al., 2020a; Tari et al., 2022). Another study with barley inclusion ranging from 0 to 56.5% found that the diets with the highest levels of barley (42.4% and 56.5%) reduced weight gain compared to a moderate inclusion level of 28.3% (Perera et al., 2019).

The grinding level of barley-based diets may influence both performance, nutrient utilization and gastrointestinal health (Perera et al., 2022). Finer grinding is generally assumed to enhance substrate availability for enzymatic digestion, while coarser grinding is considered beneficial for gizzard development (Amerah et al., 2007). In a study using barley-based diets, both coarse particle size and NSPase supplementation individually improved feed conversion ratio, but no clear interaction between the two factors was observed (Perera et al., 2020b). In the same study, NSPase supplementation did not affect digesta viscosity, regardless of barley particle size.

Efficient nutrient extraction from diets depends on an interplay between the digestive system of the broilers and their gut microbiota (Clavijo and Flórez, 2018). The gut microbiota of broilers is composed of a diverse community of microorganisms, which colonize the birds from the environment, feed and litter. The composition of the gut microbiota changes with age and is influenced by various factors, such as diet, management, and host genetics (Zhao et al., 2013). The chicken ceca have the highest density of microbes (Rychlik et al., 2023; Oakley et al., 2014), and plays a crucial role in the fermentation of undigested material. Predominant cecal microbes belong mainly to the phyla Firmicutes and Bacteroidota (Nordentoft et al., 2011; Oakley et al., 2014; Shang et al., 2018; Rychlik, 2020; Rychlik et al., 2023). The major families from the Firmicutes phylum colonizing chicken ceca include Lachnospiraceae, Ruminococcaceae, and Lactobacillaceae (Rychlik, 2020).

Several studies have shown that the chicken cecal microbiota is important for host health, nutrient metabolism, and immune system development (Brisbin et al., 2008; Stanley et al., 2014b; Oakley et al., 2014). The cecal microbiota aids in the breakdown of carbohydrates which have escaped digestion in the small intestine, and especially *Lactobacilli* from the Lactobacillaceae family are efficient carbohydrate fermenters (Rychlik, 2020). Another key function of the cecal microbiota is to produce short-chain fatty acids (SCFAs) from complex carbohydrates. These SCFAs, such as acetate, butyrate, lactate and propionate, can then be absorbed by the bird and used as an energy source (Józefiak et al., 2004). Bacteria from the family Ruminococcaceae, such as *Faecalibacterium*, are major producers of butyrate (Rychlik, 2020), a metabolite widely recognized for its health promoting effects.

In this study, we investigated the cecal microbiota and *C. perfringens* in broilers fed barley-based diets, and assessed the effects of varying degrees of grinding and supplementation of NSPase. The main objective was to evaluate whether a broiler diet including more than 50% barley would increase *C. perfringens* load and inducing shifts in the cecal microbiota composition. We hypothesize that coarse grinding and NSPase supplementation modulate the chemical properties and fermentability of high-barley diets, thereby mitigating potential increases in *C. perfringens* proliferation and alterations in cecal microbiota composition.

## Materials and methods

### Experimental setup, diets and sampling

The experimental design, including details regarding feed production and sampling procedures, were thoroughly described by Itani et al. (2025). The experiment was conducted in accordance with national regulations and guidelines of the Norwegian Food Safety Authority. According to Norwegian regulations, non-invasive feeding trials do not require a formal application for ethical approval. Day-old Ross 308 male broilers were allocated to floor pens (2.4 m x 0.95 m) bedded with wood shavings, with 22 birds per pen. From day 1 to 10, all birds were fed an identical wheat-based starter diet containing 8% barley. On day 11, the broilers were randomly assigned to different dietary treatment groups, with eight replicate pens per treatment group. In the study by Itani et al. (2025), two different feeding regimens, ad libitum or intermittent access to feed, were applied. Itani et al. (2025) investigated interactions between feeding regimen, NSPase and extent of grinding. In this study, we focused on five dietary treatment groups, all with ad libitum feed access, and conducted in-depth microbiota analyses on cecal samples, as well as investigations of effects on *C. perfringens*. These findings were considered in relation to production performance, ileal starch and protein digestibility, and jejunal digesta viscosity.

**Fig. 1:**
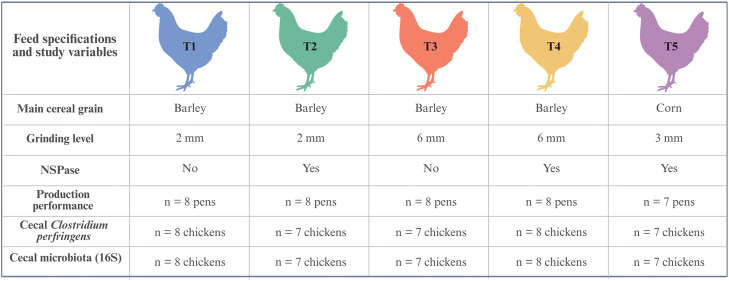
Overview of the five treatment groups (T1 – T5), including feed specifications and the number of replicates for each study variable. Created with BioRender.

Four diets were formulated using barley as the primary ingredient. These diets were characterized by the presence or absence of NSPase, as well as variations in the level of grinding of the main ingredients (2 mm or 6 mm, respectively), resulting in four treatments groups: Treatment 1 (T1) = 2 mm without NSPase, Treatment 2 (T2) = 2 mm with NSPase, Treatment 3 (T3) = 6 mm without NSPase, and Treatment 4 (T4) = 6 mm with NSPase. Furthermore, to represent a standard commercial broiler feed, a corn-based diet (grinding level 3 mm and supplemented with NSPase) was included as a control and denoted as Treatment 5 (T5). This feed was commercially produced and not specifically formulated for the study. Consequently, in addition to having a different main cereal grain, the protein and fat sources also differed from those in the barley-based diets. An overview of the five treatment groups (T1 – T5), including feed specifications and the number of replicates for each study variable, is presented in Fig. 1. All diets were steam pelleted and the feed compositions are detailed in Table 1. From day 11, the broilers were exposed to light for 16 hours a day, interrupted by two four-hour periods of darkness (10:00 pm to 02:00 am, and 03:00 am to 07:00 am).

**Table 1:** Composition and nutritional profile of the diets.

Raw material (g/kg diet)	Barley-based experimental diets (T1-T4)		Corn-based control diet (T5)
Barley*		541.1	-
Maize*		31.8	250.0
Wheat*		163.8	48.8
Soybean meal		110.9	-
Fish meal		14.4	40.0
Wheat gluten		24.1	-
Potato protein		33.7	-
Soy oil		38.5	-
Rapeseed cake meal		-	100.0
Pea meal		-	240.0
Sunflower meal		-	90.0
Animal fat		-	34.8
L-lysine HCl		3.4	3.3
DL-methionine		1.8	2.7
L-threonine		0.9	1.5
L-arginine		1.8	-
Limestone		8.7	5.2
Monocalcium phosphate (MCP)		2.1	2.5
Sodium chloride (NaCl)		4.4	0.8
Choline chloride 70%		0.7	0.6
Formic acid 85%		7.0	7.0
Titanium dioxide (TiO_2_)		5.0	-
Vitamins and minerals^1^		5.4	5.2
Phytase		0.2	0.2
NSPase^2^		0. / 0.1	0.1
*Calculated composition (g/kg diet)*			
Dry matter		894.2	872.0
Ash		46.3	42.0
Crude protein		202.0	190.1
Starch		373.5	361.2
Fat		54.0	76.7
Crude fibre		48.6	56.5
Neutral detergent fibre (NDF)		126.0	147.9
Soluble NSP		37.9	31.6
Insoluble NSP		85.4	131.3
Total non-starch polysaccharides (NSP)		123.3	162.9
Calcium		6.3	6.3
Phosphorus		4.5	5.5
Sodium		2.2	1.5
Potassium		7.0	6.8

⁎ Ingredients were ground to 2 mm for diets T1 and T2, 6 mm for diets T3 and T4, and 3 mm for diet T5.

1 Vitamin and mineral premix provided the following per kg diet: Fe 53 mg; Mn 128 mg; Zn 82 mg; Cu 15 mg; I 1 mg; Se 0.3 mg; retinyl acetate 4.1 mg; cholecalciferol 0.08 mg; dl-α-tocopheryl acetate 33 mg; menadione 4.9 mg; thiamine 3.2 mg; riboflavin 9.6 mg; niacin 48 mg; calcium pantothenate 24 mg; pyridoxine 9.6 mg; cobalamin 0.032 mg; biotin 0.3 mg; folic acid 2.6 mg.

2 Ronozyme MultiGrain (DSM Nutritional Products, Basel, Switzerland) added to diets T2 and T4, which is a thermostable multicomponent carbohydrase derived from *Trichoderma reesei*. It contains a range of different activities, of which xylanase (endo-1,4- β-xylanase; EC 3.2.1.8) and β-glucanases (endo 1,3 (4)-β-glucanase; EC3.2.1.6 and endo-1,4-β-glucanase: EC 3.2.1.4) are guaranteed activities, in addition to side activities such as xyloglucanase & arabinoxylan debranching. Diets T1 and T3 were not supplemented with NSPase. Diet T5 contained a different NSPase product.

For sample collection, eight broilers per treatment (one from each replicate pen) were euthanized during the fourth week of age. Cecal contents were collected from eight (T1 and T4) or seven (T2, T3 and T5) broilers per treatment in 1 mL LVL tubes (LVL technologies GmbH & Co, Crailsheim, Germany). The samples were immediately placed on dry ice and subsequently stored at –80°C until processing. The collection of only seven samples for some treatment groups was due to the absence of cecal contents, likely resulting from recent emptying of the ceca. The remaining broilers in the experiment were delivered to slaughter on day 35 and processed for food production. In Norway, Ross 308 broilers are typically slaughtered at 35 days of age, and this time point therefore reflects standard commercial practice.

### DNA Extraction

The DNA was extracted from cecal samples by an in-house protocol at the Norwegian Veterinary Institute. The QIAamp PowerFecal Pro DNA Kit (Qiagen, Hilden, Germany) was used following the manufacturer’s recommendations with an automated protocol on the QIAcube Connect robot (Qiagen). The samples were prepared by adding 250 mg cecal content into PowerBead Pro tubes (Qiagen). In addition, a negative control (empty tube) and a positive control containing ZymoBIOMICS Microbial Community Standard II (Zymo Research, Irvine, CA, USA) were included. Then, 800 µl CD1 buffer from the kit was added, and the samples were homogenized using FastPrep-24™ 5 G (MP Biomedicals, Solon, OH, USA) in 3 rounds x 60 sec at 5 m/s with 5 min rest between rounds. The samples were centrifuged at 15000 x *g* for 1 min and 500 µl of the supernatant was transferred to the center of the rotor adapter. The samples were extracted according to the PowerFecal Pro DNA Kit protocol with inhibitor removal on the QIAcube and eluted in 100 µl elution buffer from the kit. The concentration of the eluted DNA was measured by the Qubit dsDNA BR assay (Thermo Fisher Scientific, Waltham, MA, USA) and the purity was measured by Nanodrop (Thermo ScientificTM, Massachusetts, USA) following the manufacturer’s recommendations. The eluted DNA was stored at −20°C.

### Real-time PCR

A multiplex real-time PCR assay was performed for *C. perfringens* toxin genes *cpa* and *netB* as previously described (Granstad et al., 2021)*.* The assays targeting *cpa* and *netB* were originally developed by Albini et al. (2008) and Schlegel et al. (2012), respectively. The *cpa* gene lies within the chromosome of all *C. perfringens* strains*,* while the *netB* gene, located on plasmids, encodes a toxin associated with necrotic enteritis*.* Reactions of 25 µl included 10 x Brilliant II Probe Master Mix (Agilent Technologies, Santa Clara, California, US), dH_2_O, 20 µM primers and probes (Table 2) and 3 µl DNA extracted from the cecal samples. Temperature cycling conditions for the *C. perfringens* assay included 10 min at 50°C, 95°C for 5 min, followed by 48 cycles at 95°C for 30 s and 60°C for 1 min. Real-time PCR was performed with Bio-Rad CFX-96 real-time PCR instrument (Bio-Rad Laboratories Inc., Hercules, California, US), and data analysis was performed using Bio-Rad CFX Manager version 3.1 (Bio-Rad Laboratories Inc.). Each reaction was performed in duplicates, including a positive (*C. perfringens* strain 56, Gholamiandekhordi et al., 2006) and a negative control (nuclease-free water). The positive control was also used to generate a standard curve based on data from serial 10-fold dilutions (10°–10^−4^) to calculate the PCR efficiency. Cq values above 40 were excluded from the analysis. The assay was repeated for samples with inconsistent results. The *netB:cpa* ratios in the samples were determined by dividing the Cq values of *netB* by those of *cpa*.

**Table 2:** Primers and probes used for the real-time PCR assay of *Clostridium perfringens* toxins genes.

Target	Primer/Probe	Sequence 5′ to 3′	Reference
*cpa*	Forward Reverse Probe	AAGAACTAGTAGCTTACATATCAACTAGTGGTG TTTCCTGGGTTGTCCATTTCC HEX-TTGGAATCA-ZEN-AAACAAAGGATGGAAAAACTCAAG-IBFQ	Albini et al., 2008
*netB*	Forward Reverse Probe	GGCGGTAATATATCTGTTGAAGG ACCGTCCTTAGTCTCAAC FAM-ACTGCTGGT-ZEN-GCTGGAATAAATGCTTCA-IBFQ	Schlegel et al., 2012

### 16S rRNA amplicon sequencing

To investigate the cecal microbiota, the V3-V4 region of the 16S rRNA gene was amplified by PCR and sequenced according to recommendations from Illumina. The amplicon PCR was performed including the forward primer S-d-Bact-0341-b-S-17: CCTACGGGNGGCWGCAG and the reverse primer S-d-Bact-0785-a-A-21: GACTACHVGGGTATCTAATCC (Klindworth et al., 2013). The forward and reverse primers were tailed with partial Illumina adapters ACACTCTTTCCCTACACGACGCTCTTCCGATCT and GTGACTGGAGTTCAGACGTGTGCTCTTCCGATCT, respectively. In addition, a spacer was inserted between the gene specific sequence and the adapter as described by de Muinck et al. (2017). The primers with eight different spacers were mixed prior to PCR.

The PCR was performed with the KAPA HiFi PCR Kit (Roche, Basel, Switzerland) and 200 nM primers. The PCR was carried out on a Thermal Cycler (Thermo Fisher Scientific) with the following cycling conditions: initial denaturation at 95°C for 3 min, 25 cycles of 98°C for 20 s, 55°C for 30 s, 72°C for 30 s, and one cycle of 72°C for 10 min. The PCR products were purified with AMPure XP beads (Beckman Coulter Life Sciences, Brea, CA, USA) using a bead to sample ratio of 0.8 x.

Then, an indexing PCR was performed to attach the Illumina indices and sequencing adapters. The same PCR kit was used with 500 nM primers and the following PCR program: initial denaturation at 95°C before 8 cycles of 98°C for 30 s, 55°C for 30 s and 72°C for 30 s followed by one cycle of 72°C for 5 min. The PCR products were purified with AMPure XP beads using a bead to sample ratio of 1 x.

The purified PCR amplicons were quantified with a D1000 TapeStation kit (Agilent, Santa Clara, CA, USA) on a 4200 TapeStation System (Agilent) and the libraries were pooled in equimolar concentrations. The library pool was quantified with Qubit dsDNA HS assay (Thermo Fisher Scientific), diluted to 4 nM and denatured according to Illumina’s recommendations. The denatured library pool was spiked with 7.5% phiX Control library (Illumina, San Diego, CA, USA), and sequenced using v3 chemistry to obtain 2 × 300 bp paired reads on an Illumina MiSeq (Illumina). The raw sequencing reads were demultiplexed and the data can be accessed on the Sequence Read Archive (PRJNA1280895).

### Bioinformatics analysis

The sequences were bioinformatically analyzed by DADA2 (Callahan et al., 2016) in R version 4.2.1 (R Core Team, 2021) by following the author’s recommendations. First, primers were removed using cutadapt (Martin, 2011). The read quality profiles were inspected and the paired-end reads were trimmed using the filterAndTrim function with truncLen=*c*(270,210). The error rates of the sequences were determined and dereplicated reads were used for error correction. Then, the forward and reverse reads are merged to obtain the contig sequences that were used to construct the amplicon sequence variant (ASV) table. Chimeric sequences were removed using the command removeBimeraDenovo. Taxonomy was added down to species level to each contig using the Silva version 138.1 prokaryotic SSU training set (Quast et al., 2013) formatted for the DADA2 pipeline.

### Statistical analysis

The data on production performance, ileal starch and protein digestibility, and jejunal viscosity were assessed for normality using the Shapiro-Wilk test and for homogeneity of variance using Levene’s test. Statistical analysis of these data was performed with Stata version 18 (StataCorp LLC, 2023, College Station, Texas). Treatment groups were compared using one-way Analysis of Variance (ANOVA), followed by Tukey’s pairwise post hoc analysis. When the data were not normally distributed or did not meet the assumption of homogeneity of variance, the Kruskal-Wallis test was applied, followed by Dunn’s test with Bonferroni adjustment for multiple comparisons. Differences were considered significant at *P* < 0.05. The *C. perfringens* qPCR data was analyzed by Fisher's exact test to investigate the association between the treatments and the target genes.

For the microbial analysis, the ASV table, taxonomy and metadata were combined and further inspected in R using phyloseq version 1.42.0 (McMurdie and Holmes, 2013). All samples were downsampled to the sample with the lowest sequencing reads (95 507), resulting in 29 ASVs being removed. The rarified ASV table was used to calculate alpha and beta-diversity measures and to inspect the taxonomic diversity using phyloseq, microViz version 0.10.10 (Barnett et al., 2021) and ggplot2 version 3.4.1 (Wickham, 2016).

The alpha diversity was calculated at genus level using the ps_calc_diversity function (microViz) with the Shannon index. The Wilcoxon rank sum exact test was used to test the significance in alpha diversity of enzyme supplementation in the diets. Beta diversity was investigated on proportional data as appropriate for Bray-Curtis distances and by Nonmetric multidimensional scaling (NMDS). Ellipses were added to the ordination by the stat_ellipse (ggplot2) function with a 95% confidence level. Permutational multivariate ANOVA (PERMANOVA) was performed using the adonis2 function in vegan version 2.6-4 (Oksanen et al., 2022) on the Bray-Curtis distance matrix. Homogeneity of dispersion was tested using the betadisper test. Redundancy analysis (RDA) was performed by microViz on compositional data to assess variables associated with changes in community composition and display the top 3 genera that contribute most to the ordination axes. Variables with missing data were replaced by the means for the respective treatment group and significance was tested by ANOVA implemented in vegan with 999 permutations.

Taxonomic diversity was investigated at different taxonomic levels on relative abundances by using the comp_barplot (microViz) function. Both individual samples and merged samples for each treatment were investigated. Linear discriminant analysis Effect Size (LEfSe) was used to investigate if any taxa could explain differences between the treatments by using the run_lefse function (kw_cutoff = 0.05, lda_cutoff = 2) in the microbiomeMarker package version 1.8.0.

## Results

Data on production performance, ileal starch and crude protein digestibility, and jejunal digesta viscosity are available in Supplementary Table 1. Production performance and digesta viscosity data were normally distributed with equal variances across groups, as verified by the Shapiro-Wilk test and Levene’s test, meeting the criteria for parametric analysis with one-way ANOVA. However, digestibility data did not meet normality assumptions, and variances were unequal across groups, necessitating the use of the non-parametric Kruskal-Wallis test for these measurements. Chickens fed the control diet (T5) had a higher feed intake (*P* = 0.001) compared to those on barley-based diets (T1-T4), as shown in Table 3. However, no differences in body weight gain (BWG) or feed conversion ratio (FCR) were observed between any of the groups (*P* = 0.197 and *P* = 0.103, respectively).

**Table 3:** Mean values with standard deviations and statistics of production performance, ileal starch and protein digestibility, and jejunal viscosity by dietary treatment group.

	Production performance 11-33 days			Starch digestibility (%)	Protein digestibility (%)	Jejunal viscosity (mPa·s)
Treatment	BWG (g)	FI (g)	FCR			
T1	2333.0 a ± 98.6	3044.8 a ± 79.4	1.306 a ± 0.04	99.5 a ± 0.18	82.4 a ± 2.32	2.47 ab ± 0.35
T2	2356.3 a ± 20.2	3030.0 a ± 31.1	1.286 a ± 0.02	99.3 ab ± 0.54	81.0 a ± 3.59	2.00 a ± 0.73
T3	2321.4 a ± 94.5	3047.4 a ± 70.2	1.314 a ± 0.03	98.5 b ± 1.19	85.1 b ± 1.64	2.76 b ± 0.49
T4	2319.0 a ± 74.0	3075.8 a ± 66.4	1.327 a ± 0.04	99.3 ab ± 0.22	84.9 b ± 0.56	2.04 ab ± 0.56
T5	2407.6 a ± 79.7	3180.1 b ± 85.9	1.321 a ± 0.01	NA	NA	NA
P-value^1^	0.197	0.0013	0.103	0.085	0.017	0.0321
F/H-statistic^2^	1.60	5.66	2.09	6.62	10.19	3.40
df^3^	(4, 34)	(4, 34)	(4, 34)	3	3	(3, 27)
Post hoc adjusted p-values for pairwise comparisons^4^						
T1 vs T2	0.975	0.992	0.684	0.515	0.767	0.383
T1 vs T3	0.998	1.000	0.989	**0.016**	**0.033**	0.734
T1 vs T4	0.996	0.895	0.668	0.139	**0.024**	0.468
T1 vs T5	0.371	**0.005**	0.886	NA	NA	NA
T2 vs T3	0.899	0.986	0.393	0.071	**0.024**	**0.049**
T2 vs T4	0.875	0.673	0.082	0.402	**0.018**	0.999
T2 vs T5	0.716	**0.002**	0.206	NA	NA	NA
T3 vs T4	1.000	0.921	0.909	0.318	0.903	0.069
T3 vs T5	0.235	**0.006**	0.990	NA	NA	NA
T4 vs T5	0.212	**0.044**	0.996	NA	NA	NA

BWG = Body weight gain live birds, FI = Feed intake live birds, FCR = Feed conversion ratio (feed intake/body weight gain), mPa·*s* = millipascal-seconds, NA = Not available.

T1=2 mm, T2=2 mm:NSPase, T3=6 mm, T4=6mm:NSPase, T5=Control.

Different letters indicate significant differences between treatment groups.

1 One-way ANOVA for production performance variables and jejunal viscosity. Kruskal-Wallis for ileal starch and protein digestibility.

2 F-statistic for one-way ANOVA and H-statistic for Kruskal-Wallis.

3 Degrees of freedom.

4 Tukey’s test for production performance variables and jejunal viscosity, and Dunn’s test with Bonferroni correction for ileal starch and protein digestibility. Significant values are presented in bold.

Starch digestibility was lower (*P* = 0.048) in chickens fed the coarse barley-based diet without NSPase supplementation (T3) compared to those fed the finely ground barley-based diet without NSPase (T1), as shown in Table 3. In contrast, no difference in starch digestibility (*P* = 0.402) was observed between groups fed the fine and coarse barley-based diets with NSPase supplementation (T2 and T4). Protein digestibility was higher (*P* = 0.017) in groups fed the coarse barley-based diets (T3 and T4) compared to those on finely ground barley-based diets (T1 and T2), as detailed in Table 3. The highest viscosity in jejunal digesta was observed in chickens fed the coarse barley-based diet without NSPase (T3, Table 3). The viscosity in this group differed from the viscosity observed in the group fed the finely ground barley-based diet with NSPase (T2, *P* = 0.049).

### Real-time PCR of C. perfringens

The real-time PCR assay targeting the *C. perfringens* toxin genes revealed presence of the *cpa* gene in 43-86% of the samples within individual groups, while *netB* was detected in only 13-25% of the samples (Table 4). The *cpa* gene was detected in all groups, while *netB* was not detected in any samples from groups T2 and T5. There was no difference between the dietary treatments regarding the presence of *cpa* (*P* = 0.56) and *netB* (*P* = 0.77) in the samples. The mean Cq values were relatively high across all groups, indicating a low presence of *C. perfringens*. The *netB:cpa* ratio was similar across treatment groups in which *netB* was detected.

**Table 4:** The presence of *Clostridium perfringens* toxin genes *cpa* and *netB* as percentage of positive samples per treatment including mean Cq values, range of Cq values and the *netB:cpa* ratio.

Treatment	*cpa* (%)	Mean (*cpa*)	Range (*cpa*)	*netB* (%)	Mean (*netB*)	Range (*netB*)	*netB:cpa*
T1	63	35.4	32-38	25	33.3	31-39	0.94
T2	71	31.6	23-35	ND	ND	ND	ND
T3	86	32.8	27-35	14	32.8	32-33	0.95
T4	75	33.3	31-36	13	30.8	30-31	0.95
T5	43	33.9	33-35	ND	ND	ND	ND

T1=2 mm, T2=2mm:NSPase, T3=6 mm, T4=6mm:NSPase, T5=Control. ND=Not detected.

### Microbial diversity

**Fig. 2:**
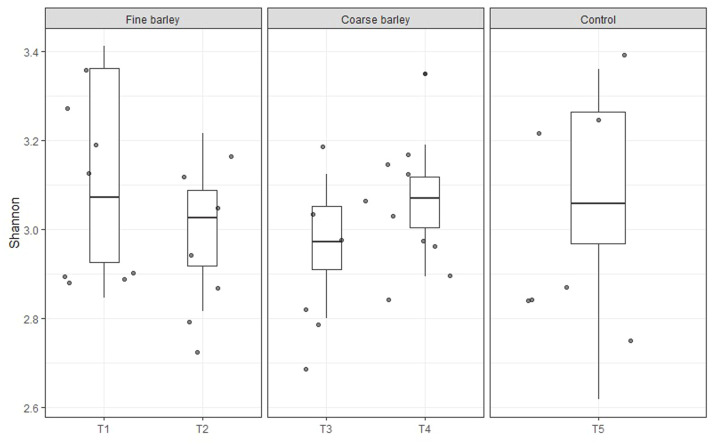
Box plots visualize alpha diversity at genus level calculated by the Shannon index in the cecal samples from broilers that received different diets grouped by grinding level. T1=2 mm, T2=2mm:NSPase, T3=6 mm, T4=6mm:NSPase, T5=Control.

We investigated the cecal microbiome in the samples from birds fed the control diet and the fine and coarse barley-based diets with and without NSPase supplementation. Similar alpha diversity was observed at genus level calculated by the Shannon index between the control and the barley-based diets (Fig. 2). There was no difference in the diversity with the supplementation of NSPase in the fine (*P* = 0.397) or the coarse (*P* = 0.189) barley-based diets.

**Fig. 3:**
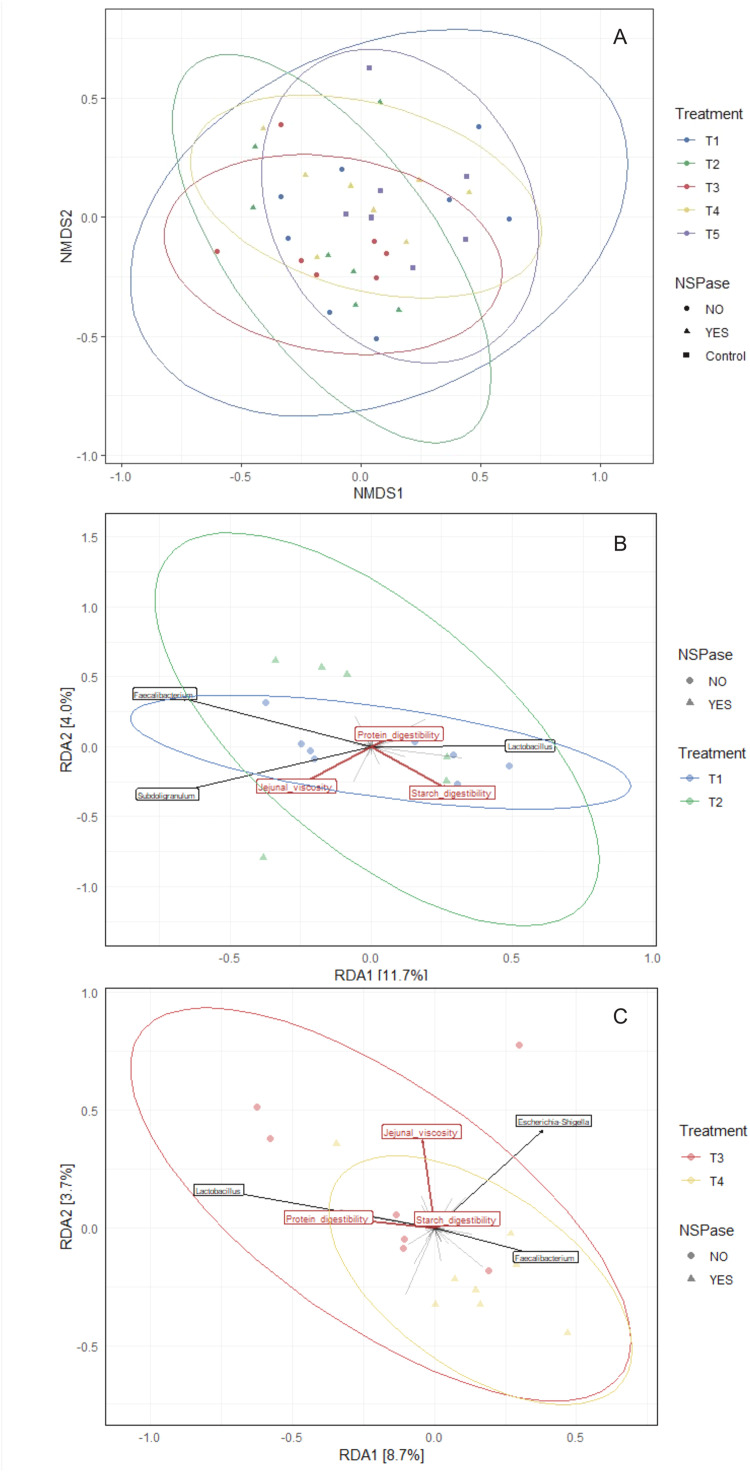
Bacterial community composition and gut health-associated variables and taxa. A) NMDS ordination plot of the bacterial community. B-C) RDA ordination plot separating the treatments by fine (B) or coarse (C) grinding including variables and genera as vectors. Each point represents one sample. The color separates the samples based on the different treatments and the shape is according to NSPase supplementation. T1=2 mm, T2=2mm:NSPase, T3=6 mm, T4=6mm:NSPase, T5=Control.

Similar beta diversity was observed between the control and the barley-based diets (Fig. 3A). The PERMANOVA adonis test detected no difference between fine and coarse grinding (*P* = 0.29), but it revealed a difference with NSPase supplementation (*P* = 0.049). No dispersion tests were significant. No difference was detected by ANOVA of the overall RDA model, including all variables, of the treatments with fine grinding (*P* = 0.845, Fig. 3B) nor coarse grinding (*P* = 0.204, Fig. 3C). However, when testing the individual variables for the coarse grinding, jejunal viscosity had an impact on the RDA (*P* = 0.045). In addition, *Lactobacillus* and *Faecalibacterium* were found to be negatively correlated.

### Taxonomic profile

**Fig. 4:**
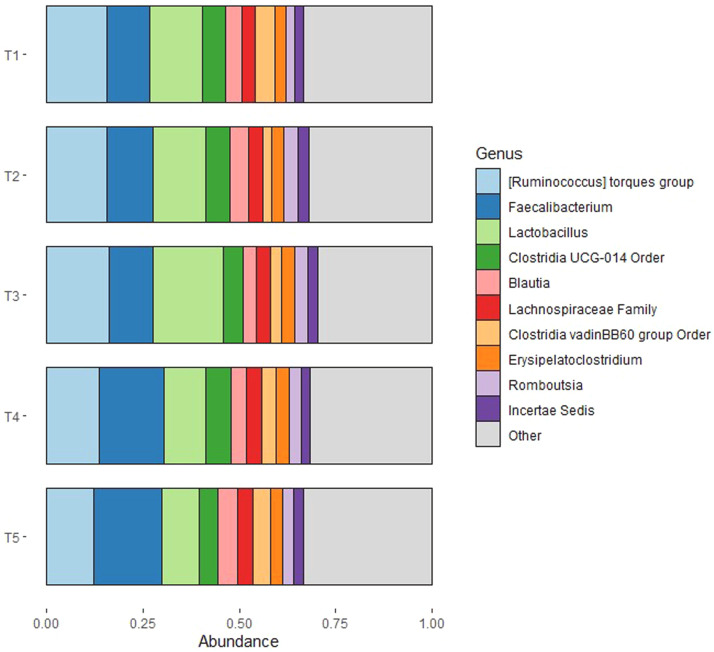
The taxonomic profile showing the relative abundance of bacteria at genus level combined by treatment. T1=2 mm, T2=2mm:NSPase, T3=6 mm, T4=6mm:NSPase, T5=Control.

The dominating phylum in all treatment groups was Firmicutes, with minor presence of Actinobacteria, Proteobacteria and Bacteriodota (Supplementary Figure 1). The major families from Firmicutes included Lachnospiraceae, Ruminococcaceae, and Lactobacillaceae (Supplementary Figure 2). The taxonomic profile was subsequently investigated at the genus level for each sample (Supplementary Figure 3) and grouped by treatment (Fig. 4). There was a substantial variation between samples within the same group. By combining the results based on treatment, the taxonomic profile at genus level was highly similar between birds fed the finely ground barley-based diets (T1-T2), regardless of NSPase supplementation. Only minor differences in *Ruminococcus* abundance were observed across treatments, with a slight decline for the groups fed the coarse diet with NSPase and the control diet. A more distinct difference in the taxonomic profile was observed among broilers fed the coarse barley-based diets (T3-T4): NSPase supplementation decreased the abundance of *Lactobacillus* and increased the abundance of *Faecalibacterium.* LEfSe associated the order Lactobacillales (*P* = 0.042, ef_lda = 3.74) and the family Lactobacillaceae (*P* = 0.046, ef_lda = 3.73) with the coarse diet without NSPase.

## Discussion

Although there was no difference in weight gain between the control group and the groups fed barley-based diets, the numerically higher weight gain in the control group may be attributed to its significantly higher feed intake. The similar feed conversion ratio across groups suggests that, although birds fed the barley-based diets consumed less feed, all groups achieved comparable growth efficiency. In the present study, the inclusion of 54% barley in broiler diets did not have a negative impact on production performance compared to a commercial broiler diet without barley. The effect of barley on production performance of broilers has been widely studied, but with varying results. Perera et al. (2019) investigated the influence of barley inclusion levels from 0 to 56.5%, with and without enzyme supplementation, on performance and nutrient utilization in broilers housed in cages. They found that the optimum inclusion level of barley based on growth performance was 28.3%, while inclusion of barley at 42.4% and 56.5% decreased feed intake and weight gain. Other recent studies have suggested that including varying amounts of barley (26-34%) in the diet does not adversely affect performance (Craig et al., 2019; Perera et al., 2020a; Tari et al., 2022). None of these studies were designed to replicate commercial conditions, where broilers are typically housed on litter, as the broilers were housed in cages or raised in pens with wire floors. This represents a strength of the present study, as the birds were kept under conditions that closely resemble commercial production systems.

Protein digestibility was higher in the groups fed the coarse barley-based diets compared to the groups fed the finely ground barley-based diets. This difference had no strong apparent effect on the cecal microbiota or *C. perfringens*, as no clear patterns distinguished the groups regardless of grinding and enzyme supplementation. Other studies have also reported higher protein digestibility in broilers fed whole or coarse barley (Tari et al., 2022; Perera et al., 2020b). Efficient protein digestion relies on prolonged retention and thorough grinding and mixing in the gizzard, which enhances contact between feed, gastric juices, and pepsin, thereby facilitating protein denaturation and breakdown (Perera et al., 2020b). In the study by Itani et al. (2025), birds fed coarse barley diets had relatively heavier gizzards than those fed finely ground barley diets (Itani et al., 2025). This suggests a more active and functional gizzard in broilers fed coarse diets, which may explain their higher ileal protein digestibility.

Barley-rich diets are not commonly used in broiler production due to the high content of NSPs and the variability in nutrient composition and quality among different barley cultivars. The increased viscosity of the digesta due to gel-forming characteristics of NSPs can impair digestion and result in a slower passage rate, promoting proliferation of bacteria like *C. perfringens* (Annett et al., 2002; Hofshagen and Kaldhusdal, 1992; Van Immerseel et al., 2004). However, the use of NSP-degrading enzymes and the development of barley cultivars have led to notable advances in overcoming previous limitations.

Our results indicate a low load of *C. perfringens* in cecal content across all groups in the study, as evidenced by high mean *cpa* gene Cq values. In Albini et al. (2008), the *cpa* gene qPCR was always able to detect 10 copies and, in some cases, even a single molecule was detected, suggesting a high level of sensitivity of the assay (Albini et al., 2008). The *netB:cpa* ratio provides an estimate of the proportion of potentially pathogenic *C. perfringens* relative to the total population in the samples. Not all samples were *netB* positive, but among the treatment groups where *netB* was detected, the *netB:cpa* ratios were consistent, suggesting that the dietary treatments did not alter the relative abundance of *netB*-positive *C. perfringens* strains. The low presence of *C. perfringens* was further supported by the 16S microbiota analysis. The two most common Clostridia in our dataset are from the UGC-014 and the Vadin BB60 groups, assigned at order level. Neither of these contain *C. perfringens*, which is assigned to the order Clostridiales, family Clostridiaceae and genus *Clostridium* in the taxonomic database used. None of the statistical analysis found differences in *Clostridium* at the genus or family level between the treatments, and the number of reads assigned to this family was low. The group fed the coarse barley-based diet without NSPase supplementation (T3) exhibited the highest viscosity of jejunal digesta. Correspondingly, this group also showed the highest presence of *C. perfringens* in the ceca, with *cpa* gene detection in 86% of samples. This association between increased digesta viscosity and higher *C. perfringens* presence aligns with findings from previous studies, which have reported that elevated viscosity can create favorable conditions for *C. perfringens* proliferation in the gut (Latorre et al., 2015; Moran, 2014). However, the *C. perfringens* loads in this study were generally very low, providing no clear indication that barley, here at 54% inclusion, predisposed the birds to development of clinical or subclinical necrotic enteritis. This contrasts with findings in other studies conducted in the 1990s and early 2000s (Kaldhusdal and Hofshagen, 1992; Riddell and Kong, 1992; Annett et al., 2002). It is possible that the hygiene and management practices in the experimental facility are maintained at a higher standard compared to those in average commercial poultry houses. Consequently, the absence of an effect of the barley-based diets on *C. perfringens* in this study can be explained by a low background population of *C. perfringens* in the broilers. Low challenge levels may limit the ability to detect statistically significant differences.

Additionally, the barley cultivar used may not have substantially promoted proliferation of *C. perfringens* due to the overall low digesta viscosity and high nutrient digestibility. Several studies, including recent work on Norwegian barley (Ahmad et al., 2025), have demonstrated considerable variation in both NSP levels and viscosity between cultivars, locations and years. Importantly, functional studies comparing low- and high-viscosity barley cultivars show that cultivar-dependent differences lead to predictable physiological effects on digesta viscosity, nutrient digestion, water intake, and *C. perfringens* proliferation, with high-viscosity cultivars showing consistently stronger negative impacts (Ahmad et al., 2025). Our findings support that NSP-related viscosity and fermentability are the main drivers of the biological responses we observed. While the strength of these effects can differ between barley cultivars, the same basic mechanisms appear to apply.

Among chickens fed the coarsely ground barley-based diet, NSPase supplementation was necessary to achieve starch digestibility similar to what was observed in chickens fed the finely ground barley-based diets. NSPase can increase starch digestibility by breaking down NSPs that encapsulate starch granules within plant cells, making them more accessible to digestive enzymes, including amylase, the enzyme responsible for starch digestion (Bedford and Schulze, 1998; Choct, 2006). For the finely ground barley-based diets, NSPase supplementation was apparently not necessary to achieve comparable starch digestibility. This can be explained by the smaller particle size increasing the exposure of starch granules to endogenous digestive enzymes, facilitating more efficient enzymatic breakdown and improving the mixing of feed particles with gut secretions.

The observed lower starch digestibility in the group fed the coarse diet without NSPase supplementation, likely caused by the increased viscosity of the digesta, coincided with a higher abundance of *Lactobacillus* in this group. In line with our findings, another study found that increased viscosity of jejunal digesta in broilers fed wheat- and barley-based diets was associated with increased cecal numbers of *Lactobacillus* (Rodríguez et al., 2012). *Lactobacillus* spp. generally ferments carbohydrates, including starch, efficiently (Rychlik, 2020). In addition, *Lactobacillus* produces metabolic compounds which decrease the pH and therefore may restrict the growth of other bacterial species (Crhanova et al., 2019). This could explain the mutual decrease of *Faecalibacterium* in this group*. Faecalibacterium* was one of the most abundant genera across treatments and has previously been found to be the most dominating bacterium in the ceca (Burrows et al., 2025). Interestingly, we observed a higher abundance of *Faecalibacterium* and no dominance of *Lactobacillus* in the group fed the coarse barley-based diet supplemented with NSPase. The addition of NSPase to the coarse barley-based diet has likely reduced the favoring of *Lactobacillus* by decreasing digesta viscosity and enhancing host starch digestibility.

In all treatment groups, *Ruminococcus* was one of the three dominant bacterial genera. This genus has been widely associated with chicken microbiota, and has been found in the respiratory tract, the intestinal tract and on eggshells, which is mainly a combination of gut and litter microbiota, feed and bedding (Rychlik et al., 2023). *Ruminococcus* is one of the most abundant bacteria in the chicken ceca (Stanley et al., 2014b). Bacteria from the family Ruminococcaceae, including *Faecalibacterium*, are major producers of butyrate from complex carbohydrates (Rychlik, 2020). Butyrate is a metabolite widely recognized for its health promoting effects and plays a key role in maintaining intestinal integrity and regulating immune responses due to strong anti-inflammatory properties (Brisbin et al., 2008; Oakley et al., 2014; Stanley et al., 2014a).

In modern commercial chicken hatcheries, high hygiene levels are maintained, and the chicks are colonized by bacteria from a variety of different sources from the environment in the poultry house rather from an adult hen (Oakley et al., 2014; Stanley et al., 2014b; Ludvigsen et al., 2016). This somewhat random and variable exposure can lead to substantial differences in the structure of gut microbiota among broiler flocks raised under different housing conditions, leading to substantial inter-trial variability (Stanley et al., 2014a). Diet is believed to be the main factor influencing the cecal microbiota (Ludvigsen et al., 2016). In our study, we observed shifts in the cecal microbiota that could be linked to the availability of starch and the level of intestinal viscosity. Coarsely ground barley appeared to benefit gizzard function and protein digestibility. However, the current study highlights the importance of NSPase supplementation in coarse barley-based diets to prevent a microbiota shift towards increased *Lactobacillus* at the expense of *Faecalibacterium*. Lactic acid bacteria have nutrient requirements resembling those of the chicken host, potentially creating competition for nutrients that might impact host performance (Apajalahti and Vienola, 2016).

It can be concluded that barley inclusion up to 54% in broiler feed with NSPase supplementation resulted in a cecal microbiota profile similar in both diversity and composition to that observed in broilers fed a commercial diet without barley, with no negative impact on performance. Furthermore, the data suggests that the risk of necrotic enteritis associated with barley inclusion in broiler diets can likely be mitigated through proper management practices, including stringent hygiene measures, thorough cleaning, and effective disinfection of the broiler house between cycles. Under a higher baseline presence of bacteria, high viscosity diets might lead to an increase in *C. perfringens*. By maintaining a low *C. perfringens* load in the environment, the potential negative effects of barley-based diets may be minimized, ensuring bird health and performance. Nevertheless, the use of NSP-degrading enzymes and the development of improved barley cultivars over the past two decades have significantly reduced the challenges associated with barley. A key strength of the present study is that the high inclusion level of this barley variety was well tolerated by the birds, supporting its use as a sustainable feed ingredient in Norwegian broiler production. Further studies are warranted to explore the effects of similar inclusion levels of different barley cultivars on gastrointestinal health and microbiota under varied environmental conditions and in slower-growing broiler hybrids.

CONFLICT OF INTEREST

The authors declare that they have no known competing financial or personal interests that could influence the work presented in this paper.

## CRediT authorship contribution statement

**Eva Lena Estensmo:** Data curation, Formal analysis, Investigation, Methodology, Writing – original draft, Writing – review & editing, Supervision. **Camilla Sekse:** Conceptualization, Project administration, Writing – review & editing. **Franciska S. Steinhoff:** Conceptualization, Funding acquisition, Writing – review & editing. **Kari Ljøkjel:** Conceptualization, Funding acquisition, Writing – review & editing. **Khaled Itani:** Conceptualization, Writing – review & editing. **Muhammad Ahmad:** Formal analysis, Writing – review & editing. **Cathrine Arnason Bøe:** Formal analysis, Writing – review & editing. **Siri Kulberg Sjurseth:** Conceptualization, Funding acquisition, Writing – review & editing. **Silje Granstad:** Conceptualization, Formal analysis, Investigation, Methodology, Project administration, Writing – original draft, Writing – review & editing.

## Disclosures

The authors declare that they have no known competing financial interests or personal relationships that could have appeared to influence the work reported in this paper.

## Appendix Supplementary materials

Image, application 1

## References

[bib0001] Ahmad M., Itani K., Ghimire S., Apajalahti J., Smith A., Steinhoff F.S., Svihus B. (2025). Effect of NSPase and feeding regimen on performance, nutrient digestibility and ileal microbiota of broiler chickens fed pelleted diets containing low- or high-viscosity barley. Anim. Feed Sci. Technol..

[bib0002] Albini S., Brodard I., Jaussi A., Wollschlaeger N., Frey J., Miserez R., Abril C. (2008). Real-time multiplex PCR assays for reliable detection of Clostridium perfringens toxin genes in animal isolates. Vet. Microbiol..

[bib0003] Amerah A.M., Ravindran V., Lentle R.G., Thomas D.G. (2007). Feed particle size: implications on the digestion and performance of poultry. World's. Poult. Sci. J..

[bib0004] Annett C.B., Viste J.R., Chirino-Trejo M., Classen H.L., Middleton D.M., Simko E. (2002). Necrotic enteritis: effect of barley, wheat and corn diets on proliferation of Clostridium perfringens type A. Avian Pathol..

[bib0005] Apajalahti J., Vienola K. (2016). Interaction between chicken intestinal microbiota and protein digestion. Anim. Feed Sci. Technol..

[bib0006] Barnett D.J., Arts I.C., Penders J. (2021). microViz: an R package for microbiome data visualization and statistics. J. Open Source Softw..

[bib0007] Bedford M.R., Schulze H. (1998). Exogenous enzymes for pigs and poultry. Nutr. Res. Rev..

[bib0008] Brisbin J.T., Gong J., Sharif S. (2008). Interactions between commensal bacteria and the gut-associated immune system of the chicken. Anim. Health Res. Rev..

[bib0009] Burrows P.B., Godoy-Santos F., Lawther K., Richmond A., Corcionivoschi N., Huws S.A. (2025). Decoding the chicken gastrointestinal microbiome. BMC Microbiol..

[bib0010] Callahan B.J., Mcmurdie P.J., Rosen M.J., Han A.W., Johnson A.J., Holmes S.P. (2016). DADA2: high-resolution sample inference from Illumina amplicon data. Nat. Methods..

[bib0011] Choct M. (2006). Enzymes for the feed industry: past, present and future. Worlds Poult. Sci. J..

[bib0012] Clavijo V., Flórez M.J.V. (2018). The gastrointestinal microbiome and its association with the control of pathogens in broiler chicken production: a review. Poult. Sci..

[bib0013] Craig A.D., Bedford M.R., Hastie P., Khattak F., Olukosi O.A. (2019). The effect of carbohydrases or prebiotic oligosaccharides on growth performance, nutrient utilisation and development of small intestine and immune organs in broilers fed nutrient-adequate diets based on either wheat or barley. J. Sci. Food Agric..

[bib0014] Crhanova M., Karasova D., Juricova H., Matiasovicova J., Jahodarova E., Kubasova T., Seidlerova Z., Cizek A., Rychlik I. (2019). Systematic culturomics shows that half of chicken caecal microbiota members can be grown in vitro except for two lineages of clostridiales and a single lineage of bacteroidetes. Microorganisms.

[bib52] de Muinck E.J., Trosvik P., Gilfillan G.D., Hov J.R., Sundaram A.Y.M. (2017). A novel ultra high-throughput 16S rRNA gene amplicon sequencing library preparation method for the Illumina HiSeq platform. Microbiome.

[bib0015] Gholamiandekhordi A.R., Ducatelle R., Heyndrickx M., Haesebrouck F., Van Immerseel F. (2006). Molecular andPhenotypical characterization of Clostridium perfringens isolatesfrom poultry flocks with different disease status. Vet. Microbiol..

[bib0016] Granstad S., Itani K., Benestad S.L., Øines Ø., Svihus B., Kaldhusdal M. (2021). Varying starch to fat ratios in pelleted diets: II. Effects on intestinal histomorphometry, Clostridium perfringens and short-chain fatty acids in Eimeria-challenged broiler chickens. Br. Poult. Sci..

[bib0017] Hofshagen M., Kaldhusdal M. (1992). Barley inclusion and avoparcin supplementation in broiler diets. 1. Effect on small intestinal bacterial flora and performance. Poult. Sci..

[bib0018] Itani K., Ahmad M., Ghimire S., Schüller R.B., Apajalahti J., Smith A., Svihus B. (2025). Interaction between feeding regimen, NSPase enzyme and extent of grinding of barley-based pelleted diets on the performance, nutrient digestibility and ileal microbiota of broiler chickens. Br. Poult. Sci..

[bib0019] Jacob J.P., Pescatore A.J. (2012). Using barley in poultry diets—a review. J. Appl. Poult. Res..

[bib0020] Józefiak D., Rutkowski A., Martin S. (2004). Carbohydrate fermentation in the avian ceca: a review. Anim. Feed Sci. Technol..

[bib0021] Kaldhusdal M., Hofshagen M. (1992). Barley inclusion and avoparcin supplementation in broiler diets. 2. Clinical, pathological, and bacteriological findings in a mild form of necrotic enteritis. Poult. Sci..

[bib0022] Klindworth A., Pruesse E., Schweer T., Peplies J., Quast C., Horn M., Glöckner F.O. (2013). Evaluation of general 16S ribosomal RNA gene PCR primers for classical and next-generation sequencing-based diversity studies. Nucleic Acids Res..

[bib0023] Latorre J.D., Hernandez-Velasco X., Kuttappan V.A., Wolfenden R.E., Vicente J.L., Wolfenden A.D., Bielke L.R., Prado-Rebolledo O.F., Morales E., Hargis B.M., Tellez G. (2015). Selection of Bacillus spp. For cellulase and xylanase production as direct-fed microbials to reduce digesta viscosity and Clostridium perfringens proliferation using an in vitro digestive model in different poultry diets. Front. Vet. Sci..

[bib0024] Ludvigsen J., Svihus B., Rudi K. (2016). Rearing room affects the non-dominant chicken cecum microbiota, while diet affects the dominant microbiota. Front. Vet. Sci..

[bib0025] Martin M. (2011). Cutadapt removes adapter sequences from high-throughput sequencing reads. EMBnet.J..

[bib0026] Mcmurdie P.J., Holmes S. (2013). phyloseq: an R package for reproducible interactive analysis and graphics of microbiome census data. PLoS One.

[bib0027] Moran E.T. (2014). Intestinal events and nutritional dynamics predispose Clostridium perfringens virulence in broilers1 1Presented in part at the Inspireform 2014, Pitlochry, Scotland. Poult. Sci..

[bib0028] Nordentoft S., Mølbak L., Bjerrum L., De Vylder J., Van Immerseel F., Pedersen K. (2011). The influence of the cage system and colonisation of Salmonella Enteritidis on the microbial gut flora of laying hens studied by T-RFLP and 454 pyrosequencing. BMC Microbiol..

[bib0029] Oakley B.B., Lillehoj H.S., Kogut M.H., Kim W.K., Maurer J.J., Pedroso A., Lee M.D., Collett S.R., Johnson T.J., Cox N.A. (2014). The chicken gastrointestinal microbiome. FEMS Microbiol. Lett..

[bib0030] Oksanen J., S. G., Blanchet F., Kindt R., Legendre P., Minchin P., O'hara R., Solymos P., Stevens M., Szoecs E., Wagner H., Barbour, M., B. M., Bolker B., Borcard D., Carvalho G., Chirico M., De Caceres M., Durand S., Evangelista H., Fitzjohn R., Friendly M., & Furneaux B., H. G., Hill M., Lahti L., Mcglinn D., Ouellette M., Ribeiro Cunha E., Smith T., Stier A., Ter Braak C., Weedon J. 2022. vegan: community ecology package. R package version 2.6-4.

[bib0031] Perera W.N.U., Abdollahi M.R., Zaefarian F., Wester T.J., Ravindran G., Ravindran V. (2019). Influence of inclusion level of barley in wheat-based diets and supplementation of carbohydrase on growth performance, nutrient utilisation and gut morphometry in broiler starters. Br. Poult. Sci..

[bib0032] Perera W.N.U., Abdollahi M.R., Zaefarian F., Wester T.J., Ravindran V. (2020). The effect of graded inclusions of waxy starch hull-less barley and a multi-component exogenous carbohydrase on the growth performance, nutrient digestibility and intestinal morphometry of broiler chickens. Br. Poult. Sci..

[bib0033] Perera W.N.U., Abdollahi M.R., Zaefarian F., Wester T.J., Ravindran V. (2020). The interactive influence of barley particle size and enzyme supplementation on growth performance, nutrient utilization, and intestinal morphometry of broiler starters. Poult. Sci..

[bib0034] Perera W.N.U., Abdollahi M.R., Zaefarian F., Wester T.J., Ravindran V. (2022). Barley, an undervalued cereal for poultry diets: limitations and opportunities. Animals.

[bib0035] Poore J., Nemecek T. (2018). Reducing food’s environmental impacts through producers and consumers. Science.

[bib0036] Quast C., Pruesse E., Yilmaz P., Gerken J., Schweer T., Yarza P., Peplies J., Glöckner F.O. (2013). The SILVA ribosomal RNA gene database project: improved data processing and web-based tools. Nucleic Acids Res..

[bib0037] R. Core Team 2021. R: a language and environment for statistical computing. R foundation for statistical computing, Vienna, Austria*.* 4.2.1 ed.

[bib0038] Riddell C., Kong X.M. (1992). The influence of diet on necrotic enteritis in broiler chickens. Avian Dis..

[bib0039] Rodríguez M.L., Rebolé A., Velasco S., Ortiz L.T., Treviño J., Alzueta C. (2012). Wheat- and barley-based diets with or without additives influence broiler chicken performance, nutrient digestibility and intestinal microflora. J. Sci. Food Agric..

[bib0040] Rychlik I. (2020). Composition and function of chicken gut microbiota. Animals.

[bib0041] Rychlik I., Karasova D., Crhanova M. (2023). Microbiota of chickens and their environment in commercial production. Avian Dis..

[bib0042] Schlegel B.J., Nowell V.J., Parreira V.R., Soltes G., Prescott J.F. (2012). Toxin-associated and other genes in Clostridium perfringens type A isolates from bovine clostridial abomasitis (BCA) and jejunal hemorrhage syndrome (JHS). Can. J. Vet. Res..

[bib0043] Shang Y., Kumar S., Oakley B., Kim W.K. (2018). Chicken gut microbiota: importance and detection technology. Front. Vet. Sci..

[bib0044] Stanley D., Geier M.S., Hughes R.J., Denman S.E., Moore R.J. (2014). Highly variable microbiota development in the chicken gastrointestinal tract. PLoS One.

[bib0045] Stanley D., Hughes R.J., Moore R.J. (2014). Microbiota of the chicken gastrointestinal tract: influence on health, productivity and disease. Appl. Microbiol. Biotechnol..

[bib0046] Svihus B., Gullord M. (2002). Effect of chemical content and physical characteristics on nutritional value of wheat, barley and oats for poultry. Anim. Feed Sci. Technol..

[bib0047] Tari L.M., Perera N., Zaefarian F., Abdollahi M.R., Cowieson A.J., Ravindran V. (2022). Influence of barley inclusion method and protease supplementation on growth performance, nutrient utilisation, and gastrointestinal tract development in broiler starters. Anim. Nutr..

[bib0048] Van Immerseel F., De Buck J., Pasmans F., Huyghebaert G., Haesebrouck F., Ducatelle R. (2004). Clostridium perfringens in poultry: an emerging threat for animal and public health. Avian Pathol..

[bib0049] Wickham H. (2016).

[bib0050] Zhao L., Wang G., Siegel P., He C., Wang H., Zhao W., Zhai Z., Tian F., Zhao J., Zhang H. (2013). Quantitative genetic background of the host influences gut microbiomes in chickens. Sci. Rep..

