# Supplementary material for: Cecal microbiota and Clostridium perfringens in broilers fed barley-based diets: Effects of enzyme supplementation and degree of grinding
Source: Poult Sci. 2026 Jan 28;105(4):106543. doi: 10.1016/j.psj.2026.106543 (PMC12919292; doi:10.1016/j.psj.2026.106543)
Supplement: Supplementary file 1 [file mmc1.docx]

Supplementary Table 1. Individual sample results for groups under ad libitum feeding conditions based on data generated by Itani et al. 2025

| Diet | | | 11-33 days | | | Ileal digestibility | | Jejunum |
| --- | --- | --- | --- | --- | --- | --- | --- | --- |
| Treatment | Grinding | NSPase | BWG | FI | FCR | Starch | Protein | Viscosity |
| T1 | 2mm | no | 2315,1 | 3019,1 | 1,304 | 99,6 | 83,6 | 2,0 |
| T1 | 2mm | no | 2250,0 | 2943,7 | 1,308 | NA | NA | 2,9 |
| T1 | 2mm | no | 2382,5 | 3012,4 | 1,264 | NA | 83,7 | NA |
| T1 | 2mm | no | 2305,9 | 3148,8 | 1,366 | 99,7 | 85,5 | 2,8 |
| T1 | 2mm | no | 2269,4 | 2950,9 | 1,300 | 99,5 | 82,2 | 2,7 |
| T1 | 2mm | no | 2292,3 | 3049,5 | 1,330 | 99,2 | 80,7 | 2,0 |
| T1 | 2mm | no | 2557,1 | 3149,2 | 1,232 | 99,6 | 82,5 | 2,5 |
| T1 | 2mm | no | 2291,6 | 3085,2 | 1,346 | 99,5 | 78,3 | 2,3 |
| T2 | 2mm | yes | 2312,8 | 3007,6 | 1,300 | 99,2 | NA | 3,3 |
| T2 | 2mm | yes | 2369,0 | 3059,8 | 1,292 | 99,8 | 83,0 | 1,5 |
| T2 | 2mm | yes | 2365,4 | 3029,9 | 1,281 | 99,7 | 76,2 | 1,8 |
| T2 | 2mm | yes | 2376,6 | 3025,7 | 1,273 | 99,4 | 78,2 | 1,4 |
| T2 | 2mm | yes | 2359,6 | 2998,9 | 1,271 | 98,2 | 83,9 | 1,6 |
| T2 | 2mm | yes | 2358,9 | 3088,6 | 1,309 | NA | NA | 2,8 |
| T2 | 2mm | yes | 2365,9 | 2998,8 | 1,268 | 99,7 | 83,9 | 1,3 |
| T2 | 2mm | yes | 2342,2 | 3030,4 | 1,294 | 99,3 | NA | 2,4 |
| T3 | 6mm | no | 2427,4 | 3055,3 | 1,259 | 98,8 | NA | 3,0 |
| T3 | 6mm | no | 2204,4 | 2978,0 | 1,351 | 99,5 | 83,7 | 3,3 |
| T3 | 6mm | no | 2222,8 | 2943,6 | 1,324 | 99,6 | 86,4 | 3,5 |
| T3 | 6mm | no | 2295,4 | 3099,4 | 1,350 | 97,5 | 82,7 | 2,2 |
| T3 | 6mm | no | 2371,3 | 3054,0 | 1,288 | 97,9 | 84,9 | 2,8 |
| T3 | 6mm | no | 2254,6 | 3011,1 | 1,336 | 96,2 | NA | 2,2 |
| T3 | 6mm | no | 2463,2 | 3166,4 | 1,285 | 98,9 | 87,1 | 2,6 |
| T3 | 6mm | no | 2331,7 | 3071,4 | 1,317 | 99,5 | 85,5 | 2,5 |
| T4 | 6mm | yes | 2422,1 | 3130,2 | 1,292 | 99,0 | NA | 2,8 |
| T4 | 6mm | yes | 2295,9 | 3025,2 | 1,318 | 99,6 | 84,1 | 2,1 |
| T4 | 6mm | yes | 2385,3 | 3149,2 | 1,320 | 99,1 | NA | 2,9 |
| T4 | 6mm | yes | 2195,5 | 3076,7 | 1,401 | 99,2 | 85,2 | 2,0 |
| T4 | 6mm | yes | 2258,5 | 2979,7 | 1,319 | 99,4 | 85,2 | 1,6 |
| T4 | 6mm | yes | 2374,8 | 3075,2 | 1,295 | 99,4 | 85,2 | 1,7 |
| T4 | 6mm | yes | 2292,9 | 3012,4 | 1,314 | 99,5 | 85,5 | 2,0 |
| T4 | 6mm | yes | 2326,7 | 3157,5 | 1,357 | 99,2 | 84,4 | 1,2 |
| T5 | NA | NA | 2432,4 | 3227,9 | 1,327 | NA | NA | NA |
| T5 | NA | NA | 2528,1 | 3267,3 | 1,292 | NA | NA | NA |
| T5 | NA | NA | 2422,7 | 3189,1 | 1,316 | NA | NA | NA |
| T5 | NA | NA | NA | NA | NA | NA | NA | NA |
| T5 | NA | NA | 2294,3 | 3037,2 | 1,324 | NA | NA | NA |
| T5 | NA | NA | 2315,6 | 3086,3 | 1,333 | NA | NA | NA |
| T5 | NA | NA | 2444,5 | 3245,4 | 1,328 | NA | NA | NA |
| T5 | NA | NA | 2415,4 | 3207,6 | 1,328 | NA | NA | NA |

BWG=Body weight gain live birds, FI=Feed intake live birds, FCR=Feed conversion ratio (feed intake/live weight gain), NA=Not available.

T1=2mm, T2=2mm:NSPase, T3=6mm, T4=6mm:NSPase, T5=Control


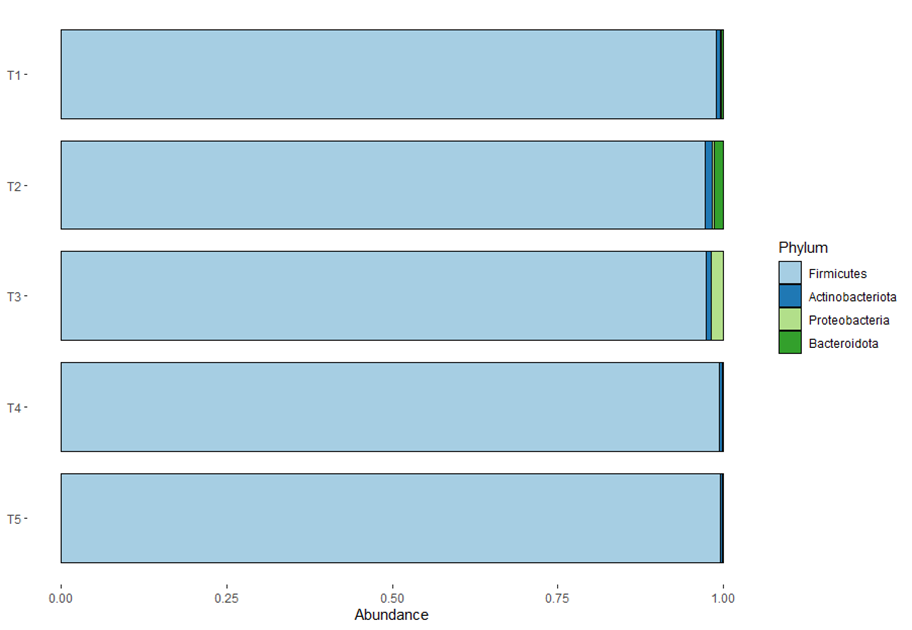
Supplementary Figure 1. The taxonomic profile showing the relative abundance at phylum level grouped by treatment: T1=2mm, T2=2mm:NSPase, T3=6mm, T4=6mm:NSPase, T5=Control.


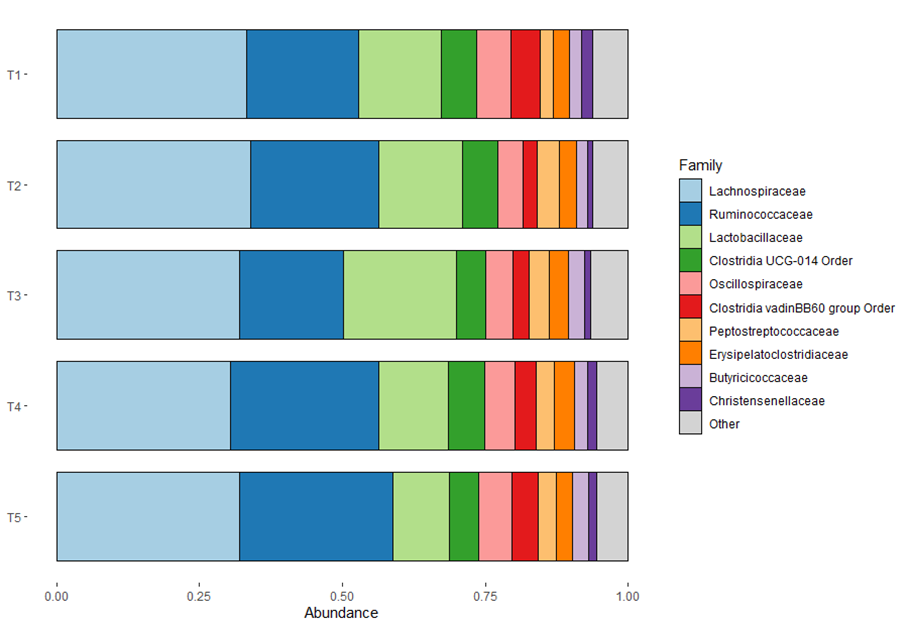
Supplementary Figure 2. The taxonomic profile showing the relative abundance at family level grouped by treatment: T1=2mm, T2=2mm:NSPase, T3=6mm, T4=6mm:NSPase, T5=Control.


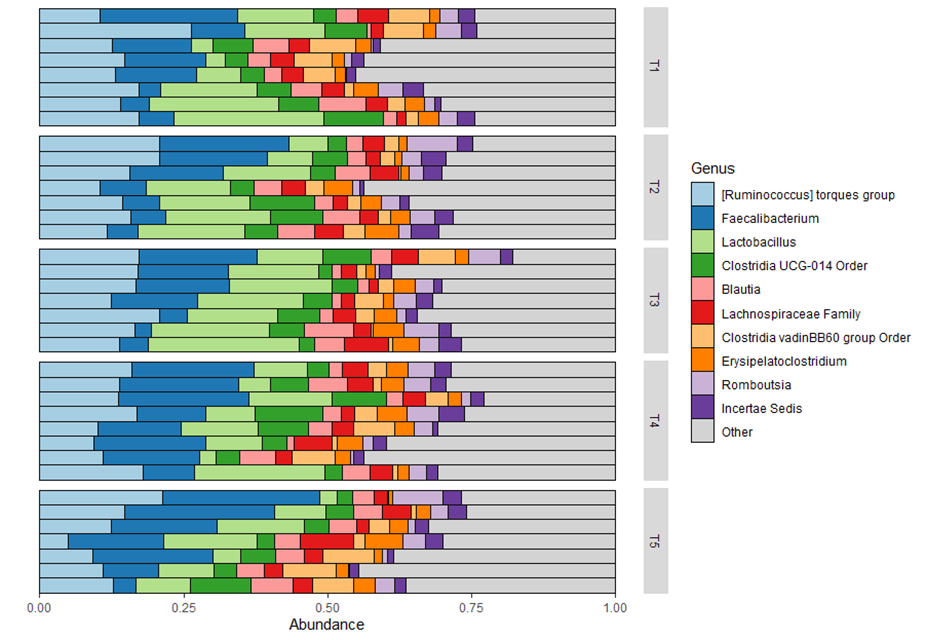


Supplementary figure 3. The taxonomic profile showing the relative abundance at genus level presented per sample. T1=2mm, T2=2mm:NSPase, T3=6mm, T4=6mm:NSPase, T5=Control.
